# Multiple quay cranes scheduling for double cycling in container terminals

**DOI:** 10.1371/journal.pone.0180370

**Published:** 2017-07-10

**Authors:** Yanling Chu, Xiaoju Zhang, Zhongzhen Yang

**Affiliations:** School of Transportation Management, Dalian Maritime University, Dalian, China; Seoul National University, REPUBLIC OF KOREA

## Abstract

Double cycling is an efficient tool to increase the efficiency of quay crane (QC) in container terminals. In this paper, an optimization model for double cycling is developed to optimize the operation sequence of multiple QCs. The objective is to minimize the makespan of the ship handling operation considering the ship balance constraint. To solve the model, an algorithm based on Lagrangian relaxation is designed. Finally, we compare the efficiency of the Lagrangian relaxation based heuristic with the branch-and-bound method and a genetic algorithm using instances of different sizes. The results of numerical experiments indicate that the proposed model can effectively reduce the unloading and loading times of QCs. The effects of the ship balance constraint are more notable when the number of QCs is high.

## 1. Introduction

As the global container trade volume continues to grow, the throughput of container terminals continues to increase as well. One of the considerations when choosing ports is the vessel turnaround time, which is mainly affected by the efficiency of quay cranes (QCs). Port managers struggle to find ways to increase operational efficiency to attract more containers in the fiercely competitive environment. Unlike other measures, such as adding equipment and terminal expansion, double cycling is a low-cost method that can efficiently improve the productivity of QCs. Due to this and its other advantages, such as increasing the utilization of trucks and QCs, double cycling has been implemented in numerous ports, such as those in Los Angeles, Norfolk and Rotterdam.

Using double cycling strategies, a QC can unload a container from the ship and load a container on the ship in the same cycle. Meanwhile, a truck can carry an outbound container to the quayside and transport an inbound container to the storage yard in one cycle. In this manner, the ship turnaround time is shortened by decreasing the empty moves of the QCs and the truck travelling distance is decreased. The benefits of double cycling can be further improved by optimizing other operations, such as the sequence of a QC working on different rows of the same bay, no-wait operation for trucks, and containers above and below deck [1]. The sequence of a QC’s activities has a direct impact on the number of double cycles and has attracted considerable attention from researchers.

However, in practical operation, there is more than one QC working on the same ship simultaneously. The scheduling of multiple QCs is more complex due to the interactions among them. In addition to non-crossing constraints, ship balance is another important factor when developing an operation plan. In particular, in double cycling strategies, the containers are unloaded by row. When an entire row is empty, the ship may lose balance to some degree. This situation may be worsened when multiple QCs are unloading the same numbered row at the same time. Therefore, scheduling the sequence of each QC jointly is a practical issue to solve.

This study addresses the multiple-QC sequencing problem with double cycling strategies. We formulate the problem as an integer programming model considering the constraint of ship balance, and a heuristic algorithm based on Lagrangian relaxation is designed to solve the model.

The remainder of this paper is organized as follows. In the next section, we review the related research. Section 3 briefly introduces the main problem and defines the mathematical formulation of the double cycling problem with the ship balance constraint. Section 4 provides a Lagrangian relaxation based heuristic and a genetic algorithm (GA) to solve it. Section 5 presents the experiments performed to test the validity of the proposed model and algorithm. We also present the performances of different solving methods and algorithms. Finally, in section 6, we draw some conclusions.

## 2. Literature review

Daganzo [2] first addressed the crane scheduling problem and formulated a linear integer programming to minimize the makespan. Many researchers subsequently began investigating QC scheduling problems to increase port productivity. Among the strategies applied in container terminals, double cycling operation has been implemented in the ports of Los Angeles, Shanghai, and Tianjin for its ability to increase the equipment utilization and productivity of QCs. Many studies have proven that its application in Ningbo Port reduced the terminal operation cost and increased the unloading and loading efficiency [3].

With the application of double cycling strategies worldwide, increasing research has been performed, including studies evaluating QC double cycling and optimizing QC scheduling problems. For example, Goodchild and Daganzo[4] evaluated the effect of double cycling strategies. They formulated the problem as a scheduling problem and provided a lower bound for all strategies. The results showed that double cycling can reduce the total loading and unloading time by 10% and the number of cycles by approximately 20%. Zhang and Kim [5] addressed the double cycling problem by considering the hatch covers when determining the operation sequence of rows in a bay. They developed a mixed-integer programming model to maximize the double cycles of QCs and used a local search-based heuristic to search for the optimal solution. Based on this research, Lee et al. [6] formulated the same problem as a two-machine flow shop scheduling problem with series-parallel precedence constraints. This problem can then be solved in polynomial time using a simplified version of Sidney’s algorithm. Goodchild and Daganzo[7] analysed the long-term impacts of double cycling on crane productivity and other operations, such as the crane operating time, number of trucks and drivers and loading plans. This paper provided a review of how double cycling strategies affect terminal operations.

Other port operations should support double cycling to increase the efficiency of QCs compared to the efficiency under traditional single-cycling operations [1]. Considerable research has addressed single-cycling QC problems, such as the work of Lee et al. [6], who proposed an efficient GA to solve the multiple-QC scheduling problem with non-crossing constraints to minimize the completion time of ships. Choo et al.[8] developed a multiple-QC model with the constraints of yard cranes and used a Lagrangian relaxation algorithm to solve the model. Karam and Eltawil [9] presents functional integration for the berth allocation, quay crane assignment and specific quay crane assignment problems. And then, Karam et al. [10] simultaneously make schedules for quay cranes and internal trucks in container terminals. Multiple-QC problems in double cycling strategies are also complex and practical. In addition to the non-crossing constraints among the QCs, the ship balance constraint is another factor affecting the QC sequence. The related research above provides references to this paper, but optimizing the multiple-QC sequence with the ship balance constraint remains an unresolved issue.

As the numbers of QCs and rows increases, the constraints and variables increase significantly. In our problem, each QC works dependently without the ship balance constraint. Because numerous researchers have considered one QC with double cycling, as stated above, we use the Lagrangian relaxation method to reach a near-optimal solution. Lagrangian relaxation [11–13]is widely used to solve large-scale mixed-integer programs. It is more efficient because difficult constraints are moved to the objective functions and become penalties. Cheung et al.[14] formulated the scheduling of the movements of cranes in a container storage yard as a mixed-integer linear program to minimize the total unfinished workload at the end of each time period and also described a Lagrangian decomposition solution procedure. Guan et al. [15] studied the crane scheduling problem considering the non-crossing constraints. A Lagrangian relaxation approach was used to obtain tight lower bounds, and a Lagrangian relaxation based heuristic was proposed to obtain a near-optimal feasible solution. Karam and ElTawil[16] propose a Lagrangian relaxation method for the integrated quay crane and internal truck assignment in container terminals.

The main contributions of our paper are as follows: (1) This paper develops a multi-crane sequencing model for double cycling strategies to minimize the makespan of the ship handling operation. The model is more practical because ship balance constraints have not been considered in double cycling problems to date. Such constraints are critical in practical operations in container terminals and affect the makespan of ships. (2) This paper solves the linear programming model with difficult constraints. An upper bound of our problem is obtained by Lagrangian relaxation and a Lagrangian relaxation algorithm is designed to obtain a feasible solution.

## 3. Model formulations

### 3.1. Problem description

**Fig 1:**
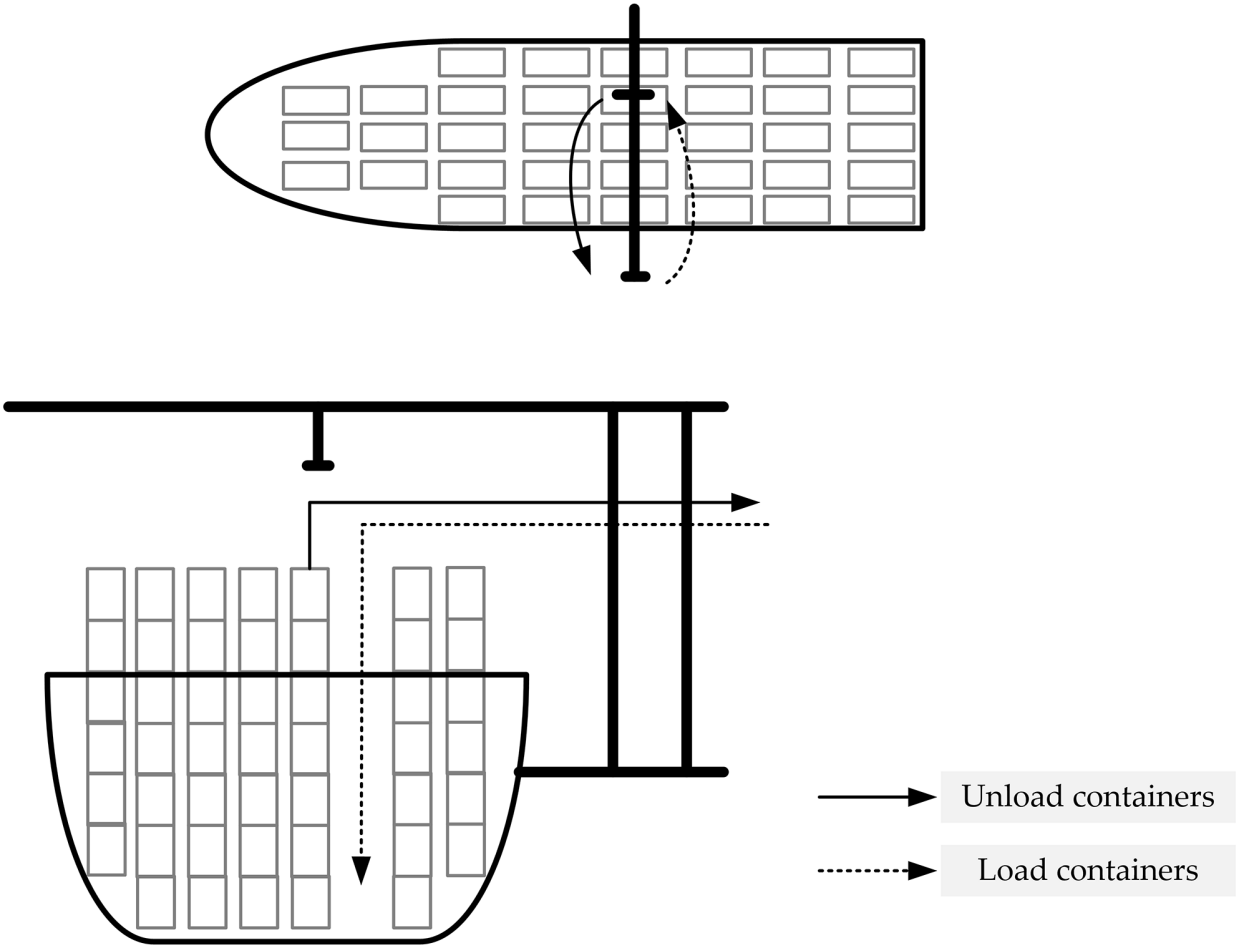
Double cycling process.

Double cycling strategies increase the efficiency of QCs because after loading a container from the quayside to the ship, a QC can unload a container from the ship to the quayside in a single cycle (as illustrated in Fig 1). Moreover, trucks can carry an inbound container from the quayside to the storage yard after carrying an outbound container from the storage yard to the quayside. Therefore, the utilization of the trucks increases, and the distance between the quayside and storage yard in the ballast decreases. However, the operation time of a QC using a double cycling strategy can be further optimized, and the sequence of each QC affects those of the others during the operation process.

#### 3.1.1. Impact of the unloading and loading sequences of QCs on completion time

**Fig 2:**
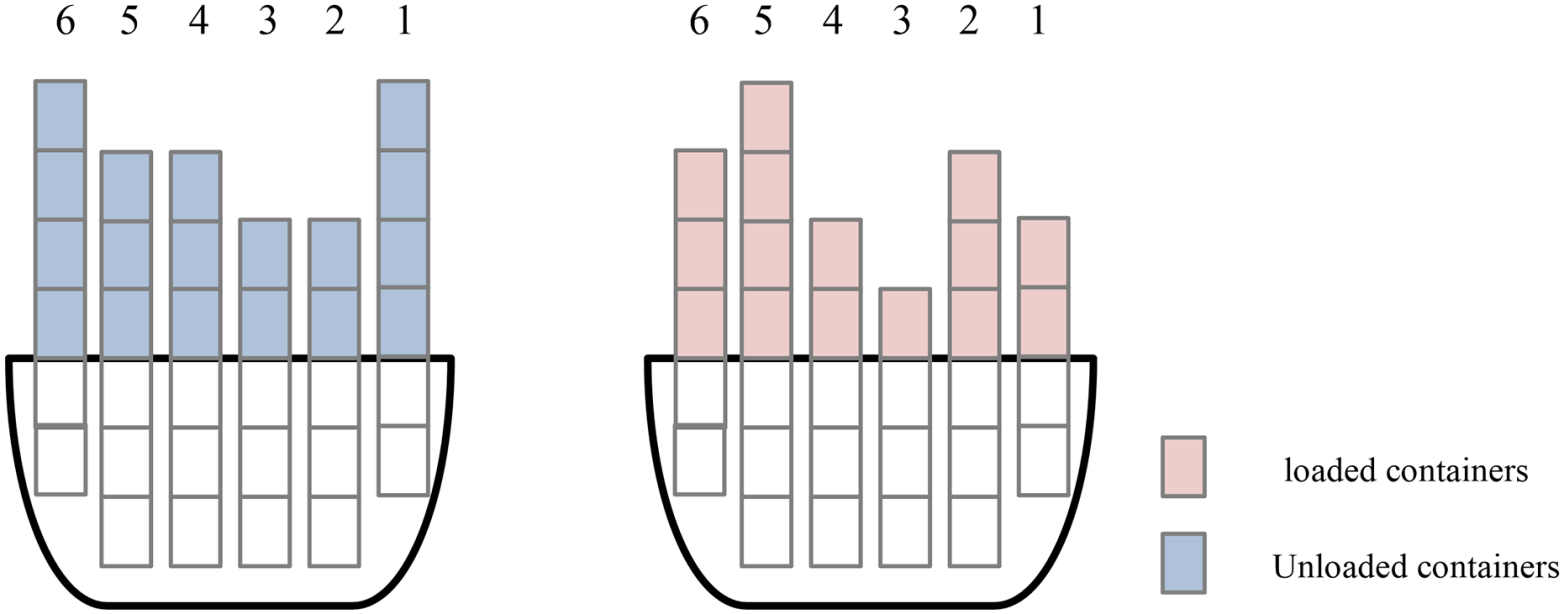
Unloading and loading plan of a ship bay.

**Fig 3:**
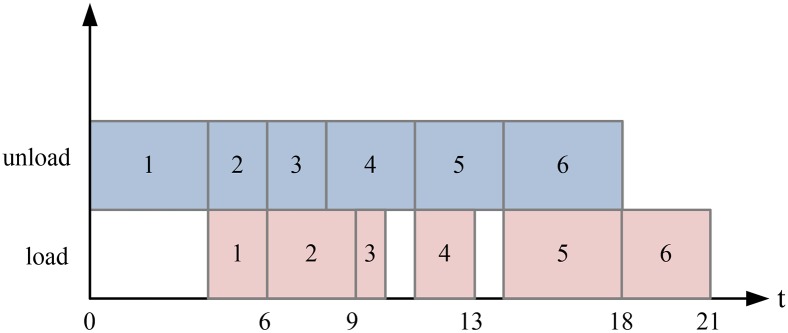
Double cycling with the sequence from quayside to seaside.

The unloading and loading plan of a ship bay is shown in Fig 2. We set a cycle from the ship to the quayside by a QC as a time unit. The rows are numbered from the quayside from 1 to 6. Fig 3 shows the total time if the QC begins unloading from 1 to 6.

**Fig 4:**
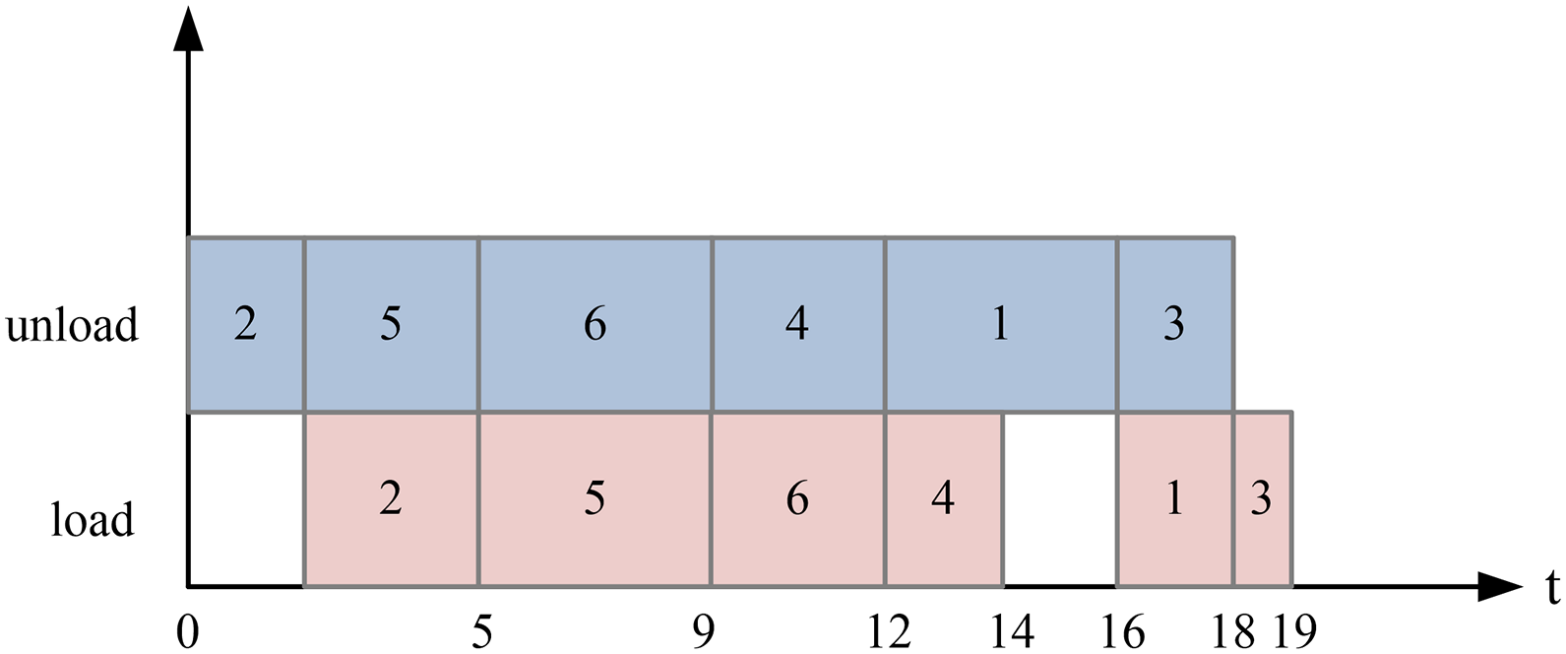
Double cycling with the sequence optimized by Johnson’s rule.

The total time in Fig 3 is 21. Fig 4 shows the operation sequence of the QC using Johnson’s rule [17] to obtain the minimum completion time. With Johnson’s rule, let Θ_1_ be a set of rows that the unloading time is smaller than the loading time. let Θ_2_ be a set of rows that the unloading time is larger than the loading time. The rows in Θ_1_ are arranged in the non-decreasing sequence of unloading time and the rows in Θ_2_ are arranged in the non-increasing sequence of loading time. Then the handling sequence are the rows in set Θ_1_ and then set Θ_2_. And If the sequence is 2-5-6-4-1-3, the total time is 19. The operation sequence affects the number of double cycles and thereby affects the total operation time. Our goal is to find the optimal operation sequence for each QC.

#### 3.1.2. Ship balance constraints

**Fig 5:**
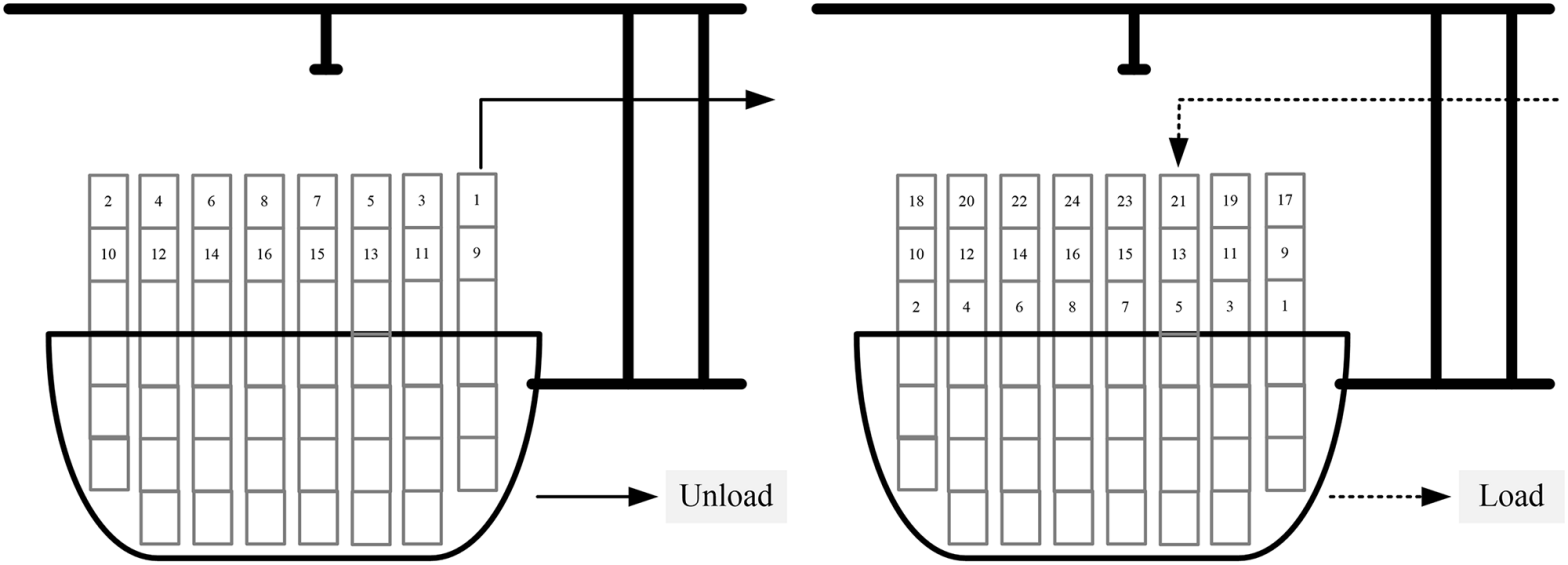
Operation sequence by tier with single cycling.

A practical factor affecting the QCs is the ship balance when unloading and loading. With a traditional operation plan, the QCs unload the ship first and then load the ship. To maintain the balance of the ship, the operation sequence is by tier, as shown in Fig 5. After unloading a container near the quayside, the QC will unload a container offshore.

**Fig 6:**
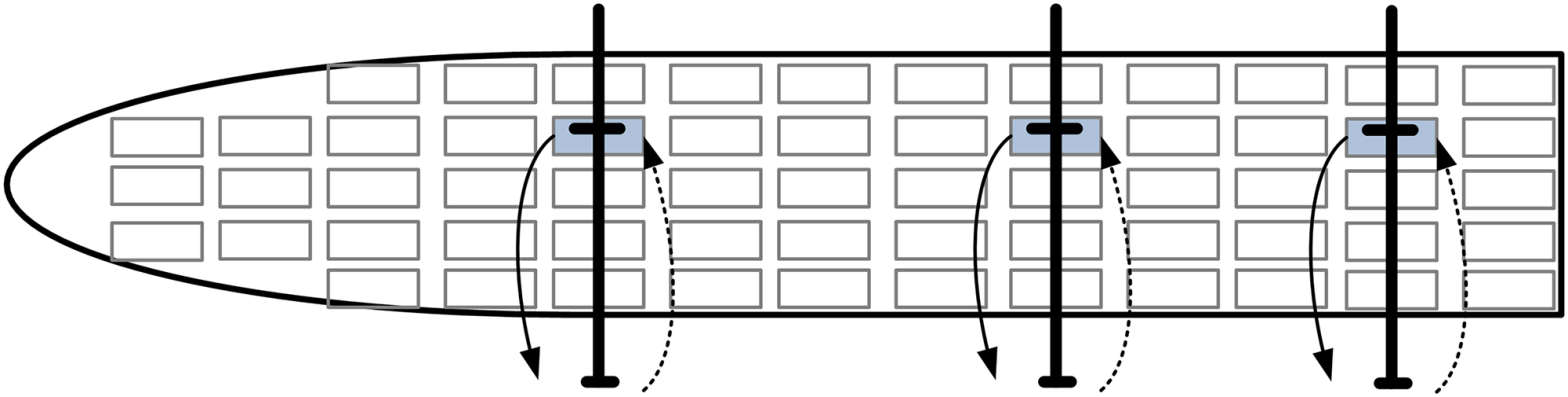
Double cycling with multiple QCs.

However, with double cycling strategies, the containers are loaded and unloaded by row. In this manner, the ship may not be balanced when one row is unloaded. Furthermore, if more QCs serve a ship simultaneously and the row numbers are close, as shown in Fig 6, the ship may become seriously unbalanced. Therefore, the issue cannot be resolved by optimizing the sequence of each QC separately. Therefore, the goal of this paper is to optimize the unloading sequence in a bay of each QC with the constraint of ship balance.

### 3.2. Mathematical formulations

In this paper, we develop a multi-crane double cycling model to optimize the operation sequence of each QC with the constraint of ship stabilization. The objective is to minimize the makespan of ship handling operation and thereby increase the double cycles of the QCs.

The following assumptions are made when formulating the model:

- The mass and locations on the ship of export and import containers are known a priori;
- The containers are all 20-feet containers;
- Trucks are sufficient to transport containers, and QCs will not wait for trucks;
- The number of QCs is known and each QC is assigned to only one bay;
- The movement of QCs between the bays is not considered;
- The QCs unload or load each container only once;
- The containers below the deck are not considered;
- The detailed information of the ship is known a priori.

We use the following sets and variables in our model:

I: the set of rows in a ship bay;

K: the set of QCs allocated to the ship;

T: the set of all time periods in the planning horizon;

*m*_*i*_,_*k*_: the mass of containers to unload from row i of bay k;

*u*_*i*_,_*k*_: the number of containers to unload from row i of bay k;

*l*_*i*_,_*k*_: the number of containers to load into row i of bay k;

*u*_*i*_,_*k*_: the number of containers to unload from row i of bay k;

*Y*_*i*_: the distance between row i and centre line of the ship;

*x*_*i*_,_*k*_,_*t*_: 1 if row i of bay k is being unloaded in the time period and 0 otherwise;

*y*_*i*_,_*k*_,_*t*_: 1 if row i of bay k is being loaded in the time period and 0 otherwise;

$t_{i,k}^{l}$: the beginning time of loading containers in row i of bay k;

$t_{i,k}^{u}$: the beginning time of unloading containers in row i of bay k;

${\tilde{t}}_{i,k}^{l}$: the completion time of loading containers in row i of bay k;

${\tilde{t}}_{i,k}^{u}$: the completion time of unloading containers in row i of bay k;

*w*_*t*_: 1 if all the containers in the ship are finished loading in time period t and 0 otherwise;

*C*: the stabilization coefficient of the ship;

Thus, the multiple-QC scheduling model is formulated as follows:

$$\text{Objective}:\;F=\text{max}\underset{t}{\sum }w_{t}$$ (1)

$$\text{s.t}.\;\underset{i}{\sum }x_{i,k,t}\leq 1,\;\forall k\in K,t\in T$$ (2)

$$\underset{i}{\sum }y_{i,k,t}\leq 1,\;\forall k\in K,t\in T$$ (3)

$$\underset{t}{\sum }x_{i,k,t}=u_{i,k},\;\forall i\in I,k\in K$$ (4)

$$\underset{t}{\sum }y_{i,k,t}=l_{i,k},\;\forall i\in I,k\in K$$ (5)

$$t_{i,k}^{l}>{\tilde{t}}_{i,k}^{u},\;\forall i\in I,k\in K$$ (6)

$${\tilde{t}}_{i,k}^{u}=t_{i,k}^{u}+u_{i,k}\text{-}1,\;\forall i\in I,k\in K$$ (7)

$${\tilde{t}}_{i,k}^{l}=t_{i,k}^{l}+l_{i,k}\text{-}1,\;\forall i\in I,k\in K$$ (8)

$$t_{i,k}^{u}=\text{min}\left\{t+M\cdot (1\text{-}x_{i,k,t})|t\in T\right\},\;\forall i\in I,k\in K$$ (9)

$$t_{i,k}^{\text{l}}=\text{min}\left\{t+M\cdot (1\text{-}y_{i,k,t})|t\in T\right\},\;\forall i\in I,k\in K$$ (10)

$${\tilde{t}}_{i,k}^{\text{u}}=\text{max}\left\{t-M\cdot (1\text{-}x_{i,k,t})|t\in T\right\},\;\forall i\in I,k\in K$$ (11)

$${\tilde{t}}_{i,k}^{\text{l}}=\text{max}\left\{t-M\cdot (1\text{-}y_{i,k,t})|t\in T\right\},\;\forall i\in I,k\in K$$ (12)

$$-C\leq \underset{i}{\sum }\underset{k}{\sum }\left(1-x_{i,k,t}\right)\cdot m_{i,k}\cdot Y_{i}\leq C,\;\forall t\in T$$ (13)

$$w_{t}\leq \frac{\underset{i}{\sum }\overset{t}{\underset{m=1}{\sum }}y_{i,k,m}}{\underset{\text{i}}{\sum }l_{i,k}},\;\forall k\in K,t\in T$$ (14)

$$w_{t},x_{i,k,t},y_{i,k,t}\in \left\{0,1\right\}$$ (15)

(Eq 1) is the objective function, which aims to minimize the competition time of the loading of all containers onto the ship. Constraints (2) and (3) ensure that one QC can only unload and load one row in any time period. Constraints (4) and (5) ensure that all of the containers in row i of bay k must be unloaded and loaded in the planning horizon. (Constraint 6) ensures that the beginning of the loading of row i must be later than the completion time of the unloading of row i. Constraints (7) and (8) provide the relationship of the completion time and beginning time of unloading and loading, respectively. Constraints (9) and (10) state the beginning times of the loading and unloading of row i of bay k, respectively. If *x*_*i*_,_*k*_,_*t*_ = 1, row i of bay k is being unloaded. Therefore, in this way, $t_{i,k}^{u}=\text{min}\left\{t+M\cdot (1\text{-}x_{i,k,t}),t\in T\right\}$ can be used to find the beginning time of loading row I of bay k. Constraints (11) and (12) state the completion times of the loading and unloading of row i of bay k, respectively. (Constraint 13) ensures the stability of the ship at any time. (Constraint 14) denotes the completion time of each row. If its right side is equal to 1 in time period t, its left side will be equal to 1 according to the objective function. (Constraint 15) denotes the binary nature of each variable.

## 4. Solution algorithms

The proposed model is an integer program that can be solved by the branch-and-bound (B&B) method. However, for a complex problem, it may not be solved in polynomial time. The Lagrangian relaxation method provides an upper bound to the original problem. The main idea is to remove complex constraints to the objective function, and then, the new problem is easy to solve. The objective of the proposed model is to optimize the sequence of the QCs with the ship balance constraint. Obviously, if the ship balance constraint is removed, each QC works independently, and several algorithms have been developed to solve the single-QC sequencing problem. Therefore, the ship balance constraint is relaxed, and then, the model is decomposed by QCs. An algorithm based on Lagrangian relaxation is designed to obtain a feasible solution. In addition, we also design a GA to solve the model to compare the efficiency of the Lagrangian relaxation based heuristic.

### 4.1. Lagrangian relaxation problem

The ship balance constraint is removed from the objective function by multiplying with Lagrangian multipliers. Then, the Lagrangian relaxation problem (LRP) becomes as follows:

$$\text{Objective}:\;F_{LR}=\text{max}\underset{t}{\sum }w_{t}+\lambda_{t}\cdot \left(\underset{i}{\sum }\underset{k}{\sum }\left(1-x_{i,k,t}\right)\cdot m_{i,k}\cdot Y_{i}-C\right)$$ (16)

Subject to Constraints (2)–(15).

*λ*_*t*_ is the Lagrangian multiplier of each constraint. Then, the LRP can be decomposed by QCs. The upper bound of the original problem can be obtained by solving the LRP. However, the quality of the upper bound depends on the multipliers. Therefore, to obtain the best upper bound, the Lagrangian multipliers should be optimized by solving the Lagrangian dual problem, which can be described as follows:

$$\text{Objective}:\;F_{\text{LD}}\left(\lambda \right)=\text{min}\underset{t}{\sum }w_{t}+\lambda_{t}\cdot \left(\underset{i}{\sum }\underset{k}{\sum }\left(1-x_{i,k,t}\right)\cdot m_{i,k}\cdot Y_{i}-C\right)$$ (17)

Subject to Constraints (2)–(15).

### 4.2. Steps of the subgradient algorithm

The subgradient method, which was first proposed by Held and Karp [18], is used to solve the Lagrangian dual problem (LRD) to search for the optimal Lagrangian multipliers. The details of the algorithm are provided below.

Step1: Choose initial Lagrangian multipliers *λ*^1^ = {0,0,…,0}, and set the iteration to 1. Then, an optimal solution can be obtained by solving the LRP. When *λ*^1^ = {0,0,…,0}, the LRP is easy to solve, as it can be decomposed by QCs. The working sequence of each QC is independent, and it can be regarded as the flow shop problem. The flow shop problem can be solved by applying Johnson’s rule [19]. Let x* represent the optimal solution of the LRP. Then, the upper bound of the original problem is the optimal objective value of the LRP, which can be described as

$$UB=F_{LR}\left(x^{*}\right)$$

If x* is feasible according to (Constraint 13), then the optimal solution of the original problem is obtained. However, if it is not feasible for the original problem, the Lagrangian relaxation based heuristic is used to adjust the solution to become feasible. Let ${\tilde{x}}^{*}$ represent a feasible solution to the original problem; then, the lower bound of the original problem can be described as

$$LB=F\left({\tilde{x}}^{*}\right)$$

If the lower bound equals the upper bound, stop the algorithm; otherwise, continue to step 2.

Step 2: Add one to the iteration, and if the iteration less than the maximum iteration, the Lagrangian multipliers should be updated as follows:

$$\lambda^{n+1}=\text{max}\{0,\lambda^{n}+t^{n}\cdot s^{n}\}$$

$$s^{n}=\sum_{i}\sum_{k}\left(1-x_{{}_{i,k,t}}^{*}\right)\cdot m_{i,k}\cdot Y_{i}$$

*s*^*n*^ is the gradient, and $x_{{}_{i,k,t}}^{*}$ is the optimal solution of the LRP. *t*^*n*^ is the step size of each iteration, which can be obtained as follows:

$$t^{n}=\frac{UB-LB}{\|s^{n}{\|}^{2}}\cdot \beta$$

At the beginning, let *β* = 2, and if the upper bound fails to change within 3 steps, the value of *β* is reduced by half.

Step 3: Continue the algorithm until the stop rules are satisfied. In this paper, the maximum iteration of the algorithm is set to 100.

### 4.3. Feasibility restoration heuristic

The Lagrangian relaxation method provides an upper bound to the original problem. In each iteration, the optimal solution of the LRP may not feasible for the original problem due to Constraint (13). This paper proposed a Lagrangian relaxation based heuristic to make an infeasible solution. The detailed steps are as follows:

Step 1: Apply Johnson’s rule to obtain the optimal sequence of each QC separately without the ship balance constraint. Johnson’s rule is a method to obtain the optimal sequence with minimum operation time. The total makespan is minimized when each QC uses the minimum operation time.
Step 2: Solve the LRP, and record the working sequence of each QC.
Step 3: For the optimal solution of the LRP, find the time that exceeds the ship balance constraint using (Eq 13).
Step 4: Choose one QC and adjust its working sequence. First, find the row number i that exceeds the ship balance constraint, and then, compare the sequence with the one optimized by Johnson’s rule in step 1. If the two sequences are the same, then adjust another QC’s sequence. Otherwise, continue to step 5.
Step 5: Find the rows that are handled before i in the sequence optimized by Johnson’s rule but after i in the sequence optimized by the LRP. We name the row set as A. For example, the sequence optimized by Johnson’s rule is 5-2-3-1-4, and the optimal sequence by the LRP is 5-1-4-3-2. If the time handling the first row is not feasible, i = 1, then the row set is A = {2, 3}.
Step 6: Choose the row has the former sequence in set A, and adjust it as the sequence optimized by Johnson’s rule. Then, the new sequence in step 5 is 5-2-1-4-3.
Step 7: Record the new total completion time, and determine whether the ship can maintain its balance during the handling. If any time exceeds the ship balance, repeat step 5 by choosing another row in set A until the sequence is feasible. If all of the rows in set A are tested and the new sequences are still infeasible, then continue to step 8.
Step 8: Choose another QC’s sequence that exceeds the ship balance constraint, and repeat step 4 until all of the QC sequences are adjusted.
Step 9: Compare the new total completion time updated in step 7. Choose the minimum completion time, and record the working sequence of QCs. The new sequence is feasible to the original problem and can be regarded as the lower bound.
Step 10: Record the new objective function value of the original problem, and compare it with the lower bound obtained in the former iteration in the subgradient algorithm. If the new lower bound is greater than the former lower bound, then the LB is updated.

Table 1 shows how to apply the Lagrangian relaxation algorithm to make a feasible solution. For each QC, two sequences are shown in two separate columns. One sequence is optimized by Johnson’s rule as step 1, and the other is the optimal sequence obtained by the LRP. Based on (Constraint 13), the time handling the 2th row in not feasible, so we need to exchange the sequence. First, we change the sequence of QC 1 using the Lagrangian relaxation based heuristic. Setting *A*_1_ = {3}, we adjust the third row to the front of the sequence. Then, the sequence of QC 1 becomes 3-4-1-2-5, and the total makespan is 129. We again determine whether the ship maintains its balance and record the new sequence. Similarly, the new sequence of QC 2 is 3-5-4-2-1, and the total makespan is 131. The sequence of QC 3 is 1-4-5-2-3, and the total makespan is 128. Because all three new sequences meet the ship balance constraint, the sequence with the minimum operation time is regarded as the lower bound.

**Table 1:** Examples using the Lagrangian relaxation based heuristic.

Quay cranes	Sequence of QC 1		Sequence of QC 2		Sequence of QC 3	
	Johnson’s rule	LRP	Johnson’s rule	LRP	Johnson’s rule	LRP
Working sequence	3	4	5	3	4	1
	1	1	4	5	5	4
	4	2	1	2	2	2
	2	5	2	1	3	3
	5	3	3	4	1	5
Makespan	121	133	116	135	117	139

After applying the proposed Lagrangian relaxation based heuristic, the adjusted solution is feasible for the original problem. Because the sequence obtained by Johnson’s rule can ensure the minimum makespan, the sequence based on it can also obtain a lower makespan. The principle of the proposed heuristic is based on this rule. Therefore, the lower bound obtained by the Lagrangian relaxation based heuristic is close to the optimal solution of the original problem.

### 4.4. GA approach

To compare the efficiency of the Lagrangian Relaxation Heuristic (LRH), a GA is also designed. The GA was first developed by Goldberg [20], and it has since been widely used in many problems. It can solve problems quickly, and the main steps are as follows:

Step 1: Population initialization. The decimal encoding method is used to represent each chromosome. A chromosome is regarded as a feasible working sequence of all QCs. Each gene represents the number of the rows. Each sequence of the QCs is separated by 0. Therefore, the length of a chromosome equals K(I+1)-1. For example, let K = 3, I = 4; then, a string of numbers 21430314204321 means that the sequence of the first QC is 2-1-4-3, the sequence of the second QC is 3-1-4-2 and the sequence of the last QC is 4-3-2-1. The feasible solution is the biggest problem of this algorithm. Therefore, the method in Section 4.3 to obtain a feasible chromosome is used.
Step 2: Fitness value. We use the objective function value as the fitness value.
Step 3: Genetic operator. New chromosomes are obtained through crossover and mutation procedures. We change the gene of each QC’s sequence independently. A crossover point is selected for every QC’s sequence. For the first parent individuals, genes are inherited before the crossover point, and for the second parent individual, genes are inherited from the first without repetition of the first parent’s inheritance. The mutation operation is also performed independently for each QC. Two random mutation points are generated and change the gene on the mutation point.
Step 4: Stopping criterion. The maximum iteration is set as the stopping criterion. The algorithm continues until the iteration reaches the maximum iteration.

## 5. Numerical experiments

This section presents the computation results optimized by the model and algorithm. First the validity of the model is tested with one example using the LRH. Then, the LRH is compared with the B&B method and GA in terms of solution quality and computational time. The B&B method was implemented using CPLEX 12.61.

### 5.1. Data collection

**Fig 7:**
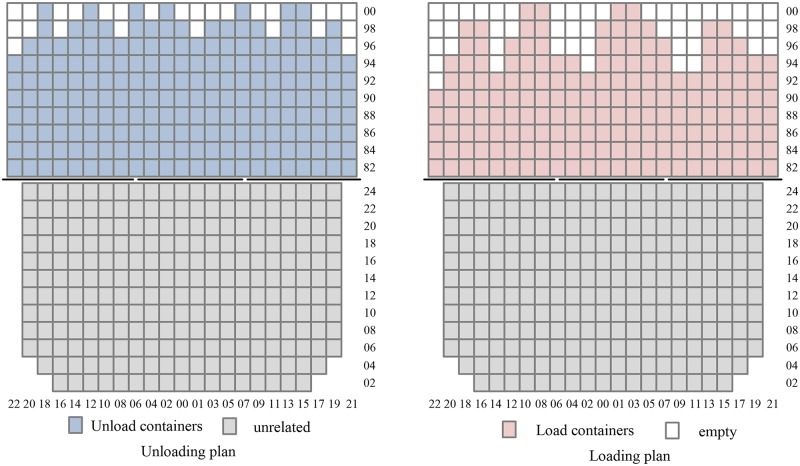
Unloading and loading plan of a bay.

One instance of a ship berthing in Tianjin Port is used to test the validity of our model. Five QCs are loading the ship simultaneously. Fig 7 shows the unloading and loading plan of one bay of the ship. There are 23 rows in a bay, and the number of tiers is 10 above the deck and 12 under the hatch. According to the stowage plan, the number of containers to unload is 203, and the number of containers to load is 182.

With double cycling strategies, a QC spends 2 minutes and 50 seconds to unload and load a container in a cycle. In contrast, with traditional single cycling, a QC spends 1 minute and 45 seconds to unload or load a container. Thus, double cycling strategies can save 40 seconds compared to single-cycling strategies.

### 5.2. Efficiency of double cycling strategies

**Fig 8:**
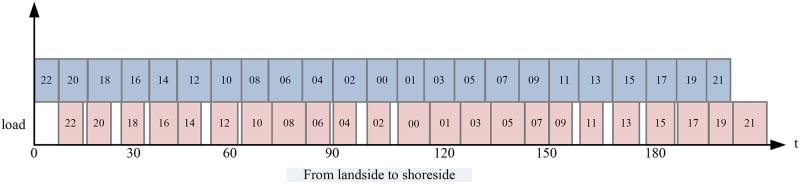
Completion time of a QC unloading from landside to quayside.

Double cycling strategies can reduce the berthing time by replacing an empty cycle of QCs with a loaded cycle. Fig 8 shows the results when the unloading operation begins from the landside to the quayside.

The total time for completing all containers with single cycling is 673.75 minutes. However, with double cycling strategies, the number of double cycles is 176, and the number of single cycles is 34. Therefore, the total time is 558.17 minutes, and the time spent is reduced by 15.58 minutes. Double cycling strategies can efficiently reduce the completion time of QCs.

### 5.3. Model validation

**Fig 9:**
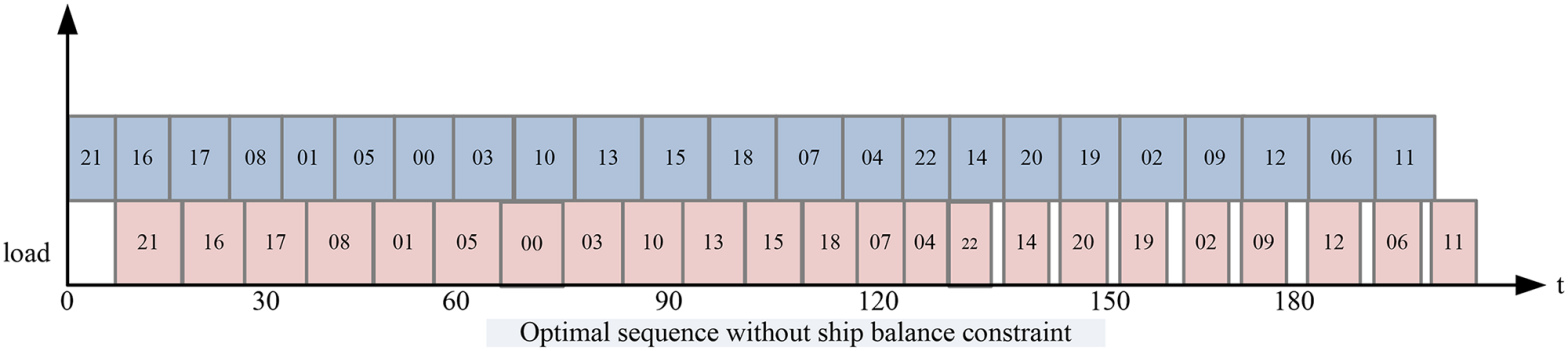
Completion time optimized with Johnson’s rule.

With double cycling, Johnson’s rule is used to optimize the sequence of the QC. The optimal working plan is shown in Fig 9.

The total completion time is 507.71 minutes after optimizing the sequence, representing a 24.6% reduction compared with traditional single cycling.

However, if there are multiple QCs working the ship simultaneously, the ship balance requirement must be satisfied. Considering the ship balance constraint, each QC may adjust its optimal sequence obtained by Johnson’s rule. Table 2 shows the completion time of five QCs and the comparison of results with and without the ship balance constraint.

**Table 2:** Results for five QCs.

QCs	Number of unloaded containers	Number of loaded containers	Makespan of single cycling (min)	Makespan of double cycling (min)	
				Without the ship balance constraint	With the ship balance constraint
QC 1	203	182	673.75	507.71	529.38
QC 2	196	173	645.75	481.17	495.57
QC 3	195	204	698.25	593.17	603.22
QC 4	212	201	722.75	587.33	614.13
QC 5	205	216	726.75	604.50	621.92
Total makespan	--	--	726.75	604.50	621.92

The completion time is 621.92 minutes when considering the ship balance constraint and 604.50 minutes without the ship balance constraint.

### 5.4. Efficiency of the LRH

Several sizes of problem are designed to test the validity of our proposed algorithms. The instances are arranged in the order of increasing QCs with varying rows in one bay. The completion time of the ship (T) depends on the number of export containers and import containers in a row. The number of import and export containers in a row is randomly distributed from 10 to 15 TEU. For small cases, we compare the efficiency of the LRH with the B&B method. In addition, we also obtain the upper bound by Lagrangian relaxation. The gap equals the difference between the value obtained by the B&B method and LRH divided by the value obtained by the B&B method. The results are shown in Table 3.

**Table 3:** Comparison of the LRH with the B&B method.

Instances	K	I	B&B		LRH		Upper bound	Gap
			Time (sec)	Value	Time (sec)	Value		
1	3	4	15.2	46	1.1	48	49	4.35%
2	3	5	39.5	39	1.3	40	44	2.56%
3	4	4	26.7	43	1.1	44	47	2.33%
4	4	5	81.3	34	1.7	36	39	5.88%
5	5	4	65.9	47	1.4	49	50	4.26%

For small instances, CPLEX can obtain the exact feasible solution in reasonable time. As the size of the problem increases, CPLEX requires a longer amount of time. The results show that the LRH can solve the model more rapidly, and the gap is under 6%. Lagrangian relaxation can provide a tight upper bound to the original problem. Therefore, the overall performance of the LRH and Lagrangian relaxation is high.

As the number of QCs and containers in each row increases, the B&B method has difficulties in obtaining an exact solution in reasonable time. Thus, to test the performance of the LRH, we design several large cases to compare the results with those obtained using the GA. The crossover rate and variation rate of the GA are set to 0.6 and 0.05, respectively. The maximum number of iterations of the GA is set to 500, and the initial population size is set to 100. The number of import and export containers in a row is randomly distributed from 18 to 25 TEU. The number of QCs ranges from 3 to 5. Each number of QCs has five different numbers of rows. As the number of rows and the workload in each row increase, the size of the time period increases significantly. Table 4 shows the numbers of constraints and variables for each instance.

**Table 4:** Problem size of each instance.

Instances	K	I	T	No. of constraints	No. of variables
1–1	3	8	238	14229	5700
1–2	3	12	338	28337	12150
1–3	3	15	413	42068	18563
1–4	3	19	513	64576	29213
1–5	3	23	613	91884	42263
2–1	4	8	238	18813	7600
2–2	4	12	338	37557	16200
2–3	4	15	413	55815	24750
2–4	4	19	513	85759	38950
2–5	4	23	613	122103	56350
3–1	5	8	238	23398	9500
3–2	5	12	338	46778	20250
3–3	5	15	413	69563	30938
3–4	5	19	513	106943	48688
3–5	5	23	613	152323	70438

For each instance, the upper bound is obtained with the Lagrangian relaxation method to evaluate the performance of the LRH and GA by comparing the objective function value. The results are shown in Table 5.

**Table 5:** Results of each instance.

Instances	Upper bound	LRH		GA	
		time (sec)	Objective value	time (sec)	Objective value
1–1	48	1.4	45	0.8	43
1–2	57	3.2	53	1.6	50
1–3	65	4.7	61	2.9	60
1–4	79	6.1	77	4.1	75
1–5	91	9.3	89	5.6	90
2–1	43	1.6	42	0.9	40
2–2	51	3.3	48	1.6	45
2–3	59	5.1	55	3.1	51
2–4	73	6.4	70	4.5	67
2–5	84	9.8	79	5.9	77
3–1	37	1.7	35	1.1	32
3–2	46	3.3	42	1.9	40
3–3	52	5.2	46	3.3	44
3–4	67	6.9	64	4.6	62
3–5	78	11.3	75	6.5	71

As the size of the problem increases, the computation time of the LRH and GA increases as well, but both are able to find an optimal solution in reasonable time. The GA can solve the model faster than the LRH, however, the benefit in terms of computation time is not obvious. From the gap compared with upper bound, the LRH can provide a solution that is closer to that of Lagrangian relaxation. The gap between the objective function value of the LRH and the upper bound is lower than that of the GA.

Experiments indicate that our proposed LRH can be used to solve the problem of different sizes in reasonable time. For small-scale cases, the solution obtained by the LRH is more similar to the exact optimal solution than the solution obtained with the GA. For large-scale cases, the LRH can solve the problem faster, and the gap with the upper bound is smaller than the gap obtained with the GA.

### 5.5. Sensitivity analysis

**Fig 10:**
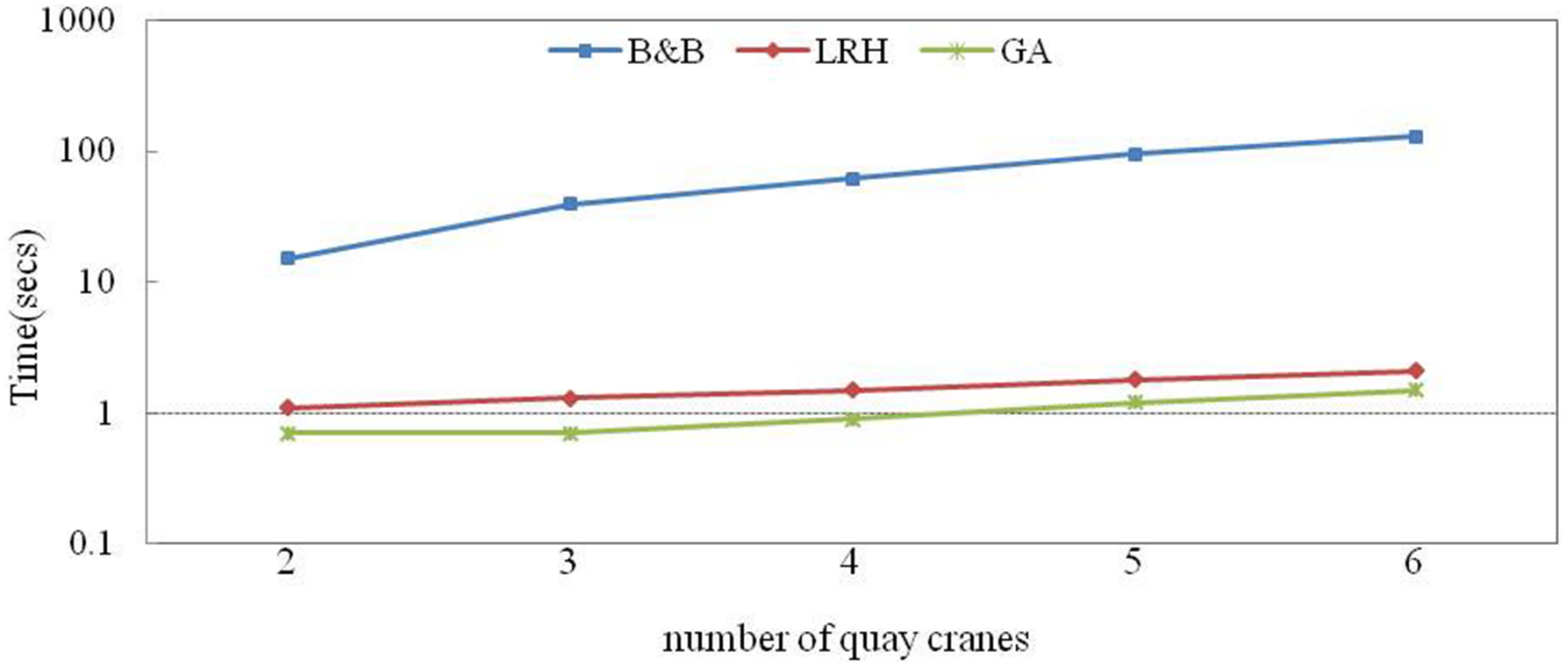
Sensitivity of the number of QCs to the computation time.

In this section, the effect of the problem size on the computation time when using the B&B method, LRH and GA is analysed. As the number of QCs, number of rows and workload increase, the constraints and variables increase rapidly. By default, we set K = 3, I = 5, and T = 300 and vary them individually. First, Fig 10 shows the computation time of the CPLEX and heuristics when the number of quays is changed from 2 to 6. The logarithmic scale is used on the Y axis because the B&B method has a long computation time. The number of QCs has a significant impact on the B&B method but a negligible effect on the LRH and GA.

**Fig 11:**
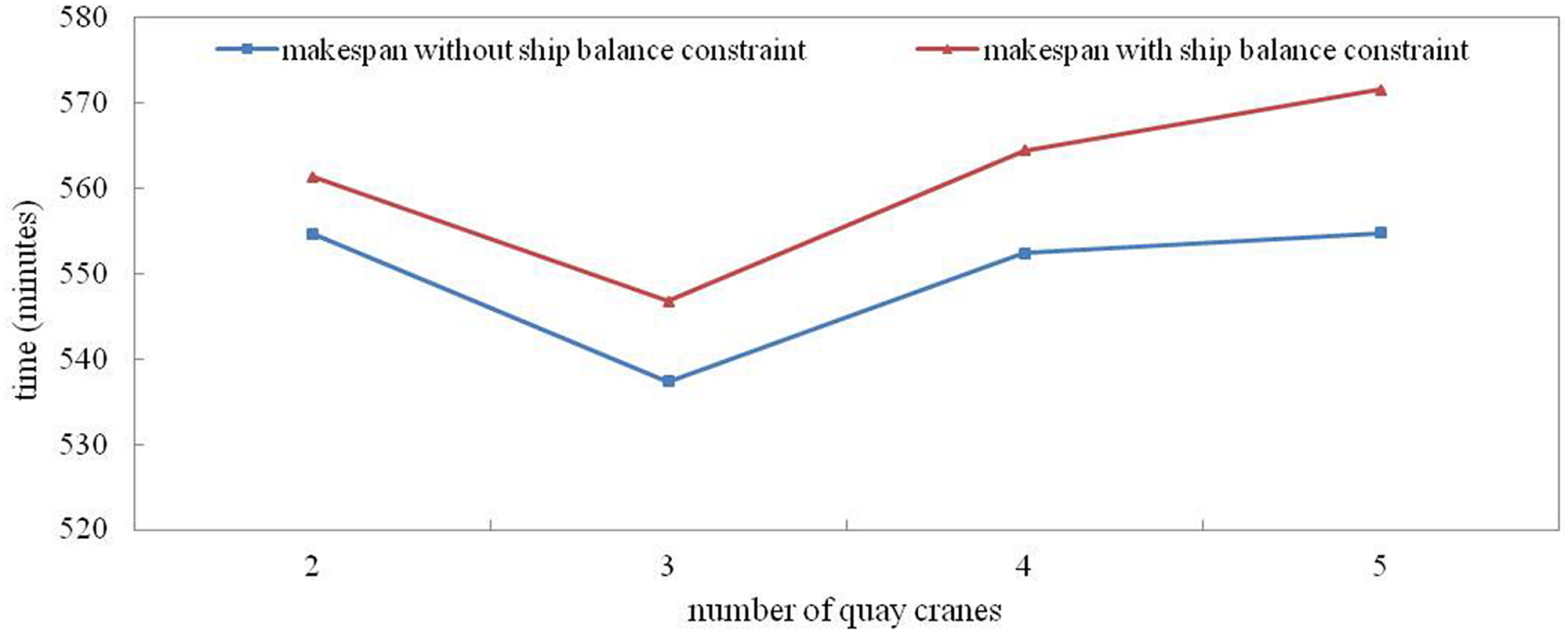
Effect of the number of QCs on the optimization result.

The difference between the two curves in Fig 11 shows the changes of makespan considering the ship balance constraint. This value becomes larger as the number of QCs increases, regardless of the average time of double cycling. This result is reasonable because the constraint increases when the number of QCs increases; thus, the sequence changes more than the optimal sequence obtained without the ship balance constraint.

**Fig 12:**
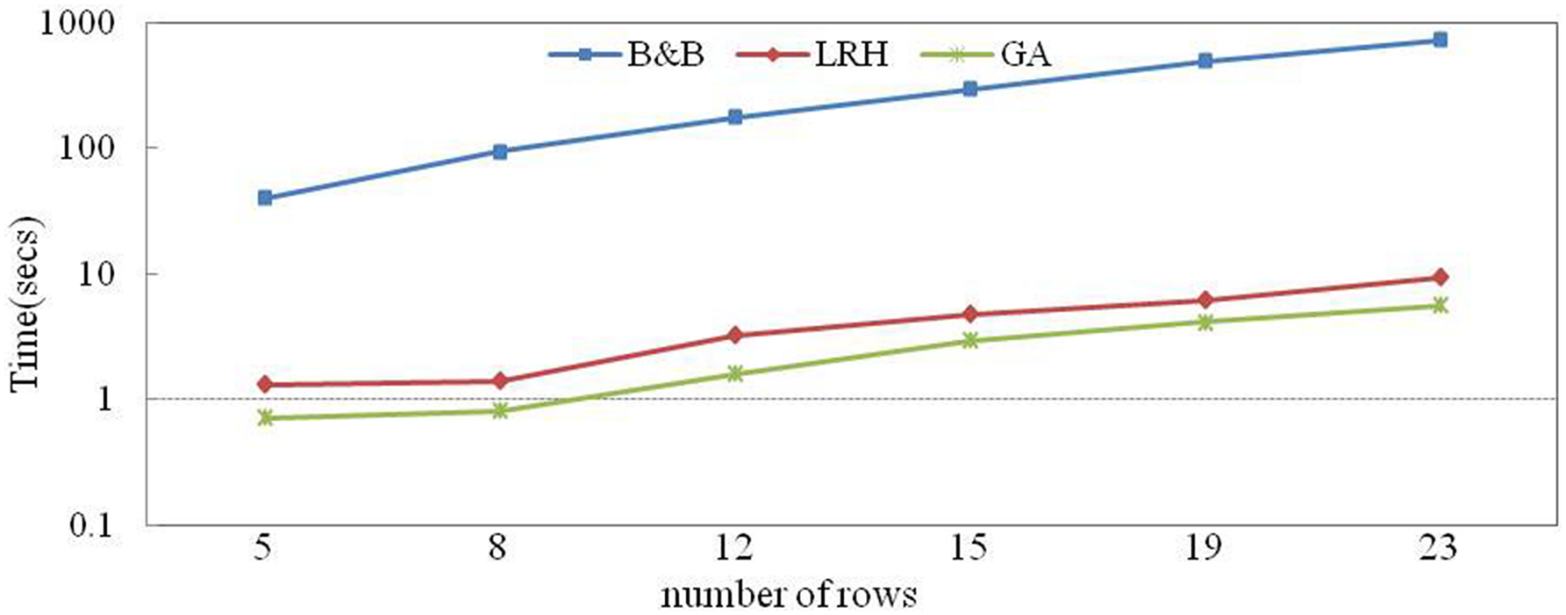
Sensitivity of the number of rows to the computation time.

Fig 12 shows the variations in computation time as the number of rows in a bay is changed from 5 to 20. The difference between the CPLEX and heuristics is more notable than in Fig 10. As the number of rows in a bay increases, the computation time of the heuristics increases only slightly, whereas the computation time of the B&B method increases rapidly.

**Fig 13:**
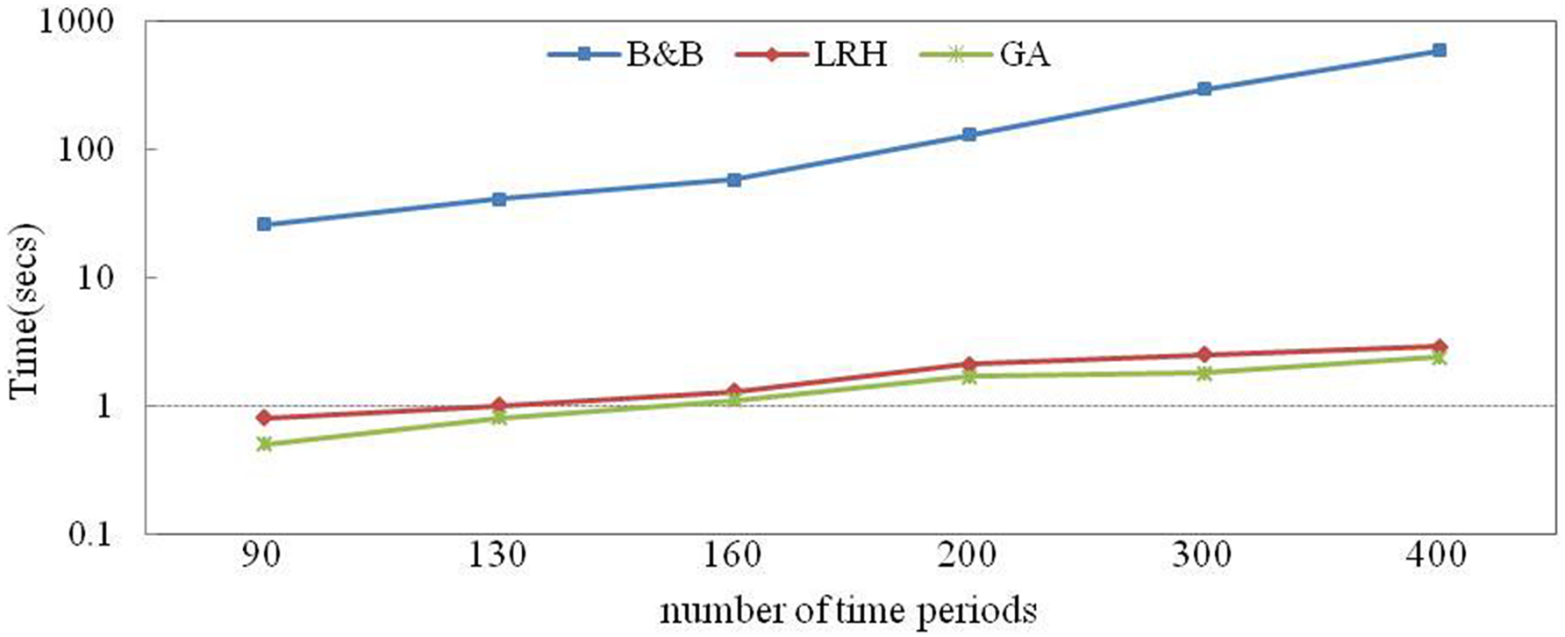
Sensitivity of the number of time periods to the computation time.

The impact of the number of periods is shown in Fig 13. The computation time of the B&B method increases more rapidly because the numbers of constraints and variables are affected by the time periods more significantly.

Changes in the numbers of crane quays, rows and time periods clearly affect the constraints and variables and thus affect the computation time. These effects are more significant for the B&B method than for the two heuristics. In addition, the computation time is robust with respect to the number of time periods.

## 6. Conclusions

In this paper, an optimization model of the sequence of multiple QCs with double cycling strategies is developed to increase the operational efficiency in a container terminal. The proposed model considers the ship balance constraint with multiple QCs unloading a ship simultaneously. Our objective is to minimize the makespan of the ship berthing. The Lagrangian relaxation method is used to obtain an upper bound of the original problem. Then, a Lagrangian relaxation algorithm is proposed to obtain a feasible solution based on the LRP. Finally, numerical experiments indicate that the optimization model can efficiently reduce the QC’s operation time, and Lagrangian relaxation can provide a tight upper bound. In addition, for small-scale problems, the B&B method can provide an exact solution in a reasonable amount of time. For large-scale problems, the performance of the Lagrangian relaxation based heuristic is also compared with that of the GA. The results show that the GA solves the problem faster, but the solution quality of the LRH is higher. The impact of the numbers of QCs, rows and time periods on the computation time of the proposed heuristics is also analysed. As expected, the computation time of the heuristics is not significantly influenced by these three parameters.

The sequence of QCs among different bays is another practical problem. It affects the makespan of the ship loading because the working time of different bays is different (Choo, 2010). However, the working time is also affected by the sequence of QCs, as described in Section 3.1. Thus, the sequence of QCs in different bays is affected. The impact of the sequence of multiple QCs should be considered in future studies.

## Supporting information

S1 File The information of each container. (PDF)
