# Supplementary material for: Multiple quay cranes scheduling for double cycling in container terminals
Source: PLoS One. 2017 Jul 10;12(7):e0180370. doi: 10.1371/journal.pone.0180370 (PMC5503256; doi:10.1371/journal.pone.0180370)
Supplement: S1 File — (PDF) [file pone.0180370.s001.pdf]

row number	the number of unload container(TEU)	the number of load containers(TEU)	the weight of unload containers(t)	the weight of load containers(t)
22	7	5	13.85	10.23
20	8	7	16.03	13.79
18	10	9	19.57	18.45
16	8	9	15.78	18.02
14	9	6	18.23	12.24
12	10	8	20.12	15.89
10	9	10	18.22	20.45
08	8	10	15.89	20.11
06	10	7	20.13	13.89
04	9	7	18.02	14.23
02	10	6	19.99	12.12
00	9	9	17.89	18.22
01	8	10	16.01	19.83
03	9	10	18.12	20.05
05	9	9	17.88	18.34
07	10	8	20.11	16.24
09	9	6	18.33	12.42
11	8	6	16.34	12.01
13	10	9	20.12	17.87
15	10	9	20.33	17.75
17	8	8	16.12	16.24
19	9	7	18.03	13.96
21	7	7	14.23	13.89
